# The ratio of serum LL-37 levels to blood leucocyte count correlates with COVID-19 severity

**DOI:** 10.1038/s41598-022-13260-8

**Published:** 2022-06-08

**Authors:** Matthias Keutmann, Gabriele Hermes, Denise Meinberger, Annika Roth, Jannik Stemler, Oliver A. Cornely, Andreas R. Klatt, Thomas Streichert

**Affiliations:** 1 Faculty of Medicine and University Hospital Cologne, University of Cologne, Institute for Clinical Chemistry, 50937 Cologne, Germany; 2 Faculty of Medicine and University Hospital Cologne, Translational Research, Cologne Excellence Cluster On Cellular Stress Responses in Aging-Associated Diseases (CECAD), University of Cologne, 50931 Cologne, Germany; 3 Faculty of Medicine and University Hospital Cologne, Department I of Internal Medicine, Center for Integrated Oncology Aachen Bonn Cologne Duesseldorf (CIO ABCD) and Excellence Center for Medical Mycology (ECMM), University of Cologne, 50937 Cologne, Germany; 4 German Centre for Infection Research (DZIF), Partner Site Bonn-Cologne, 50931 Cologne, Germany; 5 Faculty of Medicine and University Hospital Cologne, Clinical Trials Centre Cologne (ZKS Köln), University of Cologne, 50935 Cologne, Germany

**Keywords:** Prognostic markers, Viral infection

## Abstract

Beneficial effects of vitamin D on COVID-19 progression have been discussed in several studies. Vitamin D stimulates the expression of the antimicrobial peptide LL-37, and evidence shows that LL-37 can antagonize SARS-CoV-2. Therefore, we investigated the association between LL-37 and vitamin D serum levels and the severity of COVID-19. To this end, 78 COVID-19 patients were divided into 5 groups according to disease severity. We determined serum levels of LL-37, vitamin D, and routine laboratory parameters. We demonstrated a correlation of CRP, IL-6, PCT, leukocyte count, and LDH with the severity of COVID-19. Our study did not demonstrate a direct relationship between serum levels of LL-37 and vitamin D and the severity of COVID-19. LL-37 is produced by granulocytes and released at the site of inflammation. Therefore, the analysis of LL-37 in broncho-alvelolar lavage rather than in patient serum seems critical. However, since LL-37 is produced by granulocytes, we determined serum LL-37 levels as a function of leukocyte count. The LL-37/leukocyte count ratio correlates highly significantly inversely proportional with COVID-19 severity. Our results indicate that the LL-37/leukocyte count ratio could be used to assess the risk of COVID-19 progression as early as hospital admission.

## Introduction

Coronavirus disease 2019 (COVID-19) is caused by the Severe Acute Respiratory Syndrome Coronavirus 2 (SARS-CoV-2). The potential therapeutic and preventive effects of vitamin D in COVID-19 are a current topic of discussion in the media and the scientific community. Vitamin D is a fat-soluble vitamin and plays a crucial role in regulating calcium balance^1^. In addition, studies show that vitamin D has a decisive influence on the immune system and regulates antimicrobial innate immune responses^2–4^. The effects of vitamin D are mediated by its binding to the vitamin D receptor (VDR) and subsequent binding to vitamin D response elements (VREs)^2^. Such a consensus VRE is located in the promotor of the human cathelicidin antimicrobial peptide (CAMP) gene. Vitamin D stimulates the expression of the CAMP gene, which encodes for the human cathelicidin (hCap-18), the precursor of LL-37^3^. Human cathelicidin is proteolytically cleaved to LL-37 by proteinase 3 and released by exocytosis^5^. LL-37 is an antimicrobial peptide that is part of the innate immune defense. It consists of 37 amino acids that form an amphipathic, α-helical structure. At neutral pH, LL-37 is a cationic peptide with a net charge of + 6. In addition to its antibacterial properties, LL-37 has also antiviral properties^6^, and lower LL-37 serum levels are associated with severity of illness and length of hospital stay^7,8^.

SARS-CoV-2 infection is initiated by the binding of viral spike protein 1 (S1) to the host angiotensin-converting enzyme-2 (ACE2). LL-37 binds with high affinity to the receptor binding domain (RBD) of S1. LL-37 also binds to the host cell ACE2 and blocks the ligand binding domain (LBD). Both could reduce the subsequent recruitment of ACE2 and inhibit the entry of SARS-CoV-2 to the host cell^9^. Therefore, LL-37 might influence the course of COVID-19, and LL-37 serum levels might be related to infection severity.

In this study, we investigated a possible relationship between the serum levels of LL-37 and vitamin D and COVID-19 severity. In addition, we determined other laboratory parameters that might impact serum LL-37 levels or be related to COVID-19 severity.

## Material and methods

### Study design and ethics

This retrospective cohort study was performed at and under the guidelines of the University Hospital of Cologne, Germany and approved by the ethics committee of the Medical Faculty of the University of Cologne (ISI protocol, version 2.2 ‐ 02.04.2019). Written informed consent was obtained from all patients or their authorized representatives.

### Study population

The cohort enrolment criteria included patients that tested positive for SARS-CoV-2 infection by polymerase chain reaction (PCR) and were hospitalized at the University Hospital Cologne. The 78 participating patients were divided into five groups depending on the severity of the COVID-19 disease. Ten patients with asymptomatic disease course (WHO Score 1, according to the WHO Working Group on Clinical Characterization and Treatment of COVID-19 Infection^10^) formed the first group. The second group consisted of 13 patients who were symptomatic but did not require oxygen administration during hospitalization (WHO Score 2–4). The third group included 26 patients whose COVID-19 disease required non-invasive oxygen administration (WHO Score 5 or 6). The fourth group comprised 13 patients who required invasive mechanic tube ventilation (WHO Score 7–9). The fifth group included 16 patients who died of COVID-19 during the hospitalization (WHO Score 10).

### Blood samples

Blood samples were collected between day 1 and day 3 of clinical admission of each individual inpatient stay between December 2020 and March 2021. Serum was collected in 4.7-ml serum monovettes (Sarstedt, Germany), centrifuged at 2772 g for 10 min, and stored at -80 °C until use. Ethylendiamintetraazetat (EDTA) blood was collected in EDTA monovettes and used for hematological analysis (Sarstedt, Germany).

### Enzyme-linked immunosorbent binding assay (ELISA)

To detect LL-37, sandwich ELISA was performed with a Human Antibacterial Protein LL-37 ELISA Kit from Abbexa® strictly following the provided protocol. In brief, 96 well plates were coated with an anti-LL-37 antibody. LL-37 standards and patient serum were added to the wells, and a biotin-conjugated reagent was added to the wells and incubated. Unbound conjugates were removed using the provided wash buffer at each stage. Tetramethylbenzidine 6 (TMB 6) substrate was used to quantify the HRP enzymatic reaction. Optical density (OD) was measured spectrophotometrically at 450 nm. Samples of each patient were measured in duplicate.

### Other laboratory measurements

C-reactive protein (CRP) was determined by the CRP Latex Test Gen. 3, a particle-enhanced immunoturbidimetry (Roche Diagnostics®) with the Cobas C702 analyzer system. Interleukin 6 (IL-6) was determined by the Elecsys IL-6 (Roche Diagnostics®), a sandwich immunoassay, using the Cobas E801 analyzer system. Procalcitonin (PCT) was determined using the Elecsys BRAHMS PCT sandwich immunoassay (Roche Diagnostics), with the Cobas E801 analytical system (Roche Diagnostics). Lactate dehydrogenase (LDH) concentration was measured by a simple optical assay of the IFCC (International Federation of Clinical Chemistry and Laboratory Medicine) method using the Cobas C702 analyzer system. The white blood cells were determined from EDTA blood using the Sysmex XN-9100 or XN1000®. The concentration of 25-hydroxycholecalciferol (25-HCC, Calcidiol) was determined using Elecsys Vitamin D total II (Roche Diagnostics®), a competitive binding assay, with the Cobas E801 analytical system. 1,25-Dihydrocholecalciferol (1,25-DHCC, Calcitriol) was determined using a sandwich assay with chemiluminescence in the LIAISON® immunoassay analyzer (DiaSorin). Creatinine concentration was measured using the Creatinine Plus Test Ver. 2 (Roche Diagnostics®), an enzymatic method, on the Cobas C702. Immunoglobulins A (IgA), G (IgG), and M (IgM) were determined by Tina-quant assay (Roche Diagnostics®), an immunoturbidimetry, using the Cobas C702 analytical system. Aspartate aminotransferase (ASAT) and alanine aminotransferase (ALAT) were detected using the ASAT test and ALAT test (Roche Diagnostics®), a coupled optical test of the 7 IFCC method, on the Cobas C702. The concentration of gamma-glutamyl transferase (gamma-GT) in heparin plasma was determined using the gamma-glutamyl transferase test (Roche Diagnostics®) of the IFCC method, a color test. Apolipoproteins were analyzed using Tina-quant immunoturbidimetry (Roche Diagnostics®) with the Cobas C702 analyzer system. The concentration of triglycerides was determined by Triglycerides GPO-PAP (Roche Diagnostics®) using the Cobas C 702 analytical system. Cholesterol concentration, low-density lipoprotein (LDL) and high-density lipoprotein (HDL) were measured using the enzymatic color test Cholesterol Oxidase-Phenol 4-Aminoantipyrine Peroxidase (CHOD-PAP) (Roche Diagnostics®) on the Cobas C702.

### Statistical analysis

Descriptive statistics are presented with average value, standard deviation, median, and interquartile range. Normality of distribution was checked with Kolmogorov–Smirnov criterion and graphically with histograms. Relations between parameters were expressed as correlation coefficients Spearman’s Rho for quantitative factors. The statistical analysis was performed using IBM SPSS Statistics 28.0.0.0. (IBM Corp., Armonk, NY, USA). All p-values were two-tailed with p < 0.05 considered statistically significant.

### Ethics approval

All methods were carried out in accordance with relevant guidelines and regulations.

## Results

### Demographics

**Figure 1:**
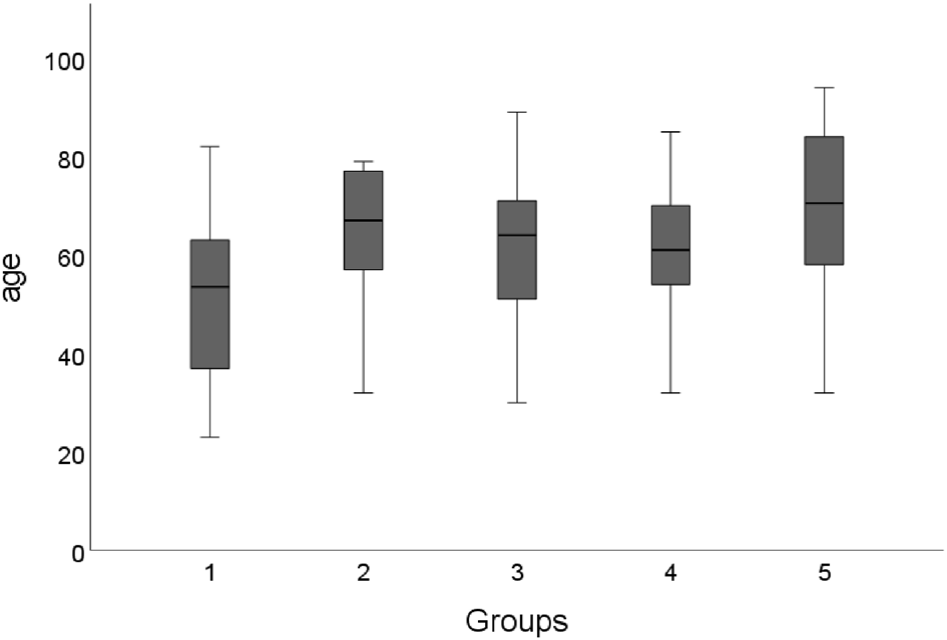
Boxplot of age and severity of COVID-19. The age distribution of the five groups. The median, upper quartile, lower quartile, minimum and maximum are shown.

The patient ages in our study ranged from 23 to 94 years (Fig. 1). The mean age was 62 years, with a standard deviation of 17 years (Table 1). The mean age was highest in group 5 at 69 years. There was no statistically significant difference between the groups.

**Table 1:** Clinical characteristics and laboratory data. The clinical characteristics and laboratory data obtained in the study. The mean values of the groups and the reference ranges are listed. Values below the reference range are marked with a minus, values above with a plus.

	Group 1	Group 2	Group 3	Group 4	Group 5	Total average	Reference range
Severity of COVID-19	Asymptomatic	Mild symptoms	Not invasively ventilated	Mechanical ventilated	Deceased		
WHO score	1	2–4	5–6	7–9	10		
N	10	13	26	13	16	78	
Age	52	63	61	60	68	62	
Male : female	7:3	6:7	13:13	8:5	8:8	42:36	
LL-37 [ng/ml]	86.6	71.2	64.7	57.4	68.9	68.2	
LL-37/leukocyte count	81	20	12	8	8	21	
LL-37/lymphocyte count	99	94	113	61	112	99	
25-HCC [µg/l]	− 20	− 21	− 26	− 16	− 22	− 22	30–70
1,25-DHCC [ng/l]	43.8	36.9	48.3	51.4	48.3	46.5	19.9–79.3
CRP [mg/l]	+ 59.2	+ 61.0	+ 115.2	+ 92.2	+ 155.6	+ 102.6	< 5.0
IL-6 [ng/l]	+ 38.9	+ 35.9	+ 90.0	+ 284.3	+ 291.1	+ 148.4	< 8.0
Procalcitonin [µg/l]	+ 0.1	+ 0.1	+ 0.5	+ 0.7	+ 1.0	+ 0.6	< 0.1
LDH [U/l]	+ 276	+ 264	+ 358	+ 446	+ 333	+ 355	< 250
Leukocytes [× 10^9^/l]	4.7	6.6	7.6	9.2	11.0	10.5	4.4–11.3
Lymphocyte count [× 10^9^/l]	1.2	− 1.1	1.3	1.2	− 0.7	− 1.1	1.2−3.5
IgG [g/l]	10.3	11.0	9.7	12.1	10.4	10.5	7.0–16.0
IgA [g/l]	1.9	2.2	2.3	2.9	2.2	2.3	0.7–4.0
IgM [g/l]	1.1	1.1	0.9	1.4	1.0	1.1	0.4–2.3
ASAT [U/I]	+ 59 (m) 26 (f)	49 (m) + 51 (f)	+ 50 (m) + 65 (f)	+ 95 (m) + 79 (f)	+ 56 (m) + 38 (f)	+ 56 (m) + 55 (f)	< 50 (m) < 35 (f)
ALAT [U/I]	+ 58 (m) 18 (f)	+ 55 (m) + 38 (f)	46 (m) + 35 (f)	+ 126 (m) + 133 (f)	23 (m) 26 (f)	+ 60 (m) + 46 (f)	< 50 (m) < 35 (f)
Gamma-GT [U/I]	+ 78 (m) + 42 (f)	+ 98 (m) + 51 (f)	+ 181 (m) + 73 (f)	+ 382 (m) + 387 (f)	+ 80 (m) + 102 (f)	+ 174 (m) + 116 (f)	< 60 (m) < 40 (f)
Creatinine [mg/dl]	+ 1.5 (m) 0.6 (f)	+ 2.8 (m) 0.6 (f)	+ 1.6 (m) + 1.3 (f)	1.1 (m) + 1.2 (f)	+ 1.5 (m) + 1.0 (f)	+ 1.6 (m) + 1.0 (f)	0.5–1.1 (m) 0.5–0.9 (f)
Apolipoprotein A-1 [mg/dl]	118 (m) − 106 (f)	− 94 (m) 113 (f)	− 87 (m) − 91 (f)	− 72 (m) − 92 (f)	− 84 (m) − 106 (f)	− 90 (m) − 100 (f)	104–202 (m) 108–225 (f)
Apolipoprotein B [mg/dl]	105 (m) 108 (f)	97 (m) 98 (f)	106 (m) 83 (f)	94 (m) 88 (f)	79 (m) 95 (f)	97 (m) 91 (f)	66–133 (m) 60–117 (f)
Apoliporotein B/A-1	1.01	0.95	1.30	1.61	1.16	1.31	
Triglycerides [mg/dl]	174	164	172	+227	152	176	< 200
Cholesterol [mg/dl]	185	163	155	158	156	161	< 200
HDL [mg/dl]	− 38 (m) − 33 (f)	− 30 (m) − 39 (f)	− 32 (m) − 34 (f)	− 23 (m) − 29 (f)	− 35 (m) 46 (f)	− 32 (m) − 37 (f)	> 40 (m) > 45 (f)
LDL [mg/dl]	121	103	101	76	92	98	< 150
Chol./HDL-Chol. quotient	+ 5.5	4.9	+ 6.7	+ 19.4	5.0	+ 8.0	< 5.1

We investigated the association between sex and COVID-19 severity because previous studies have shown an association^11,12^. Our study population consisted of 42 (54%) male and 36 (46%) female patients (Supplementary Table 1). No association was found.

### Clinical characteristics and laboratory data

The patients were clinically classified into five groups according to the severity of COVID-19 disease, as described. Serum LL-37 and vitamin D levels were determined to investigate a potential correlation between serum LL-37 and vitamin D levels and COVID-19 severity. In addition, other laboratory parameters that might be related to serum LL-37 levels and the severity of COVID-19 were determined (Table 1).

The correlation between the individual parameters and the COVID-19 severity was calculated as Spearman's rank correlation (Table 2). A correlation coefficient of 1 describes a perfect positive correlation, whereas a correlation coefficient of − 1 describes a perfect negative correlation. A correlation is significant at *p* < 0.05 for two-sided tests.

**Table 2:** Correlation of quantitative factors with the severity of COVID-19. The Spearman correlation of each parameter with COVID-19 severity. Listed are the correlation coefficients and p-values. Significant differences are shown in bold.

Parameter	Coefficient	*p*-value	Parameter	Coefficient	*p*-value	Parameter	Coefficient	*p*-value
Age	0.177	0.121	LDH	**0.244**	**0.032**	Gamma-GT	0.191	0.096
Sex	0.038	0.744	Leukocyte count	**0.344**	**0.002**	Apo_A1	− 0.218	0.055
LL-37	− 0.130	0.257	Lymphocyte count	− 0.181	0.152	Apo_B	− 0.142	0.215
LL-37/leukocyte count ratio	**− 0.373**	** < 0.001**	Creatinin	0.131	0.252	Apo_B/A-1	0.160	0.266
LL-37/lymphocyte count ratio	0.058	0.650	IgG	0.084	0.473	Triglyceride	0.009	0.939
Calcitriol	− 0.026	0.834	IgA	0.145	0.212	Cholesterin	− 0.161	0.163
Calcidiol	− 0.059	0.607	IgM	− 0.037	0.746	HDL	− 0.058	0.612
CRP	**0.340**	**0.003**	ASAT	− 0.098	0.395	LDL	**− 0.248**	**0.030**
IL-6	**0.470**	** < 0.001**	ALAT	− 0.127	0.268	Chol./HDL	− 0.034	0.770
Procalcitonin	**0.404**	**0.002**						

### LL-37

**Figure 2:**
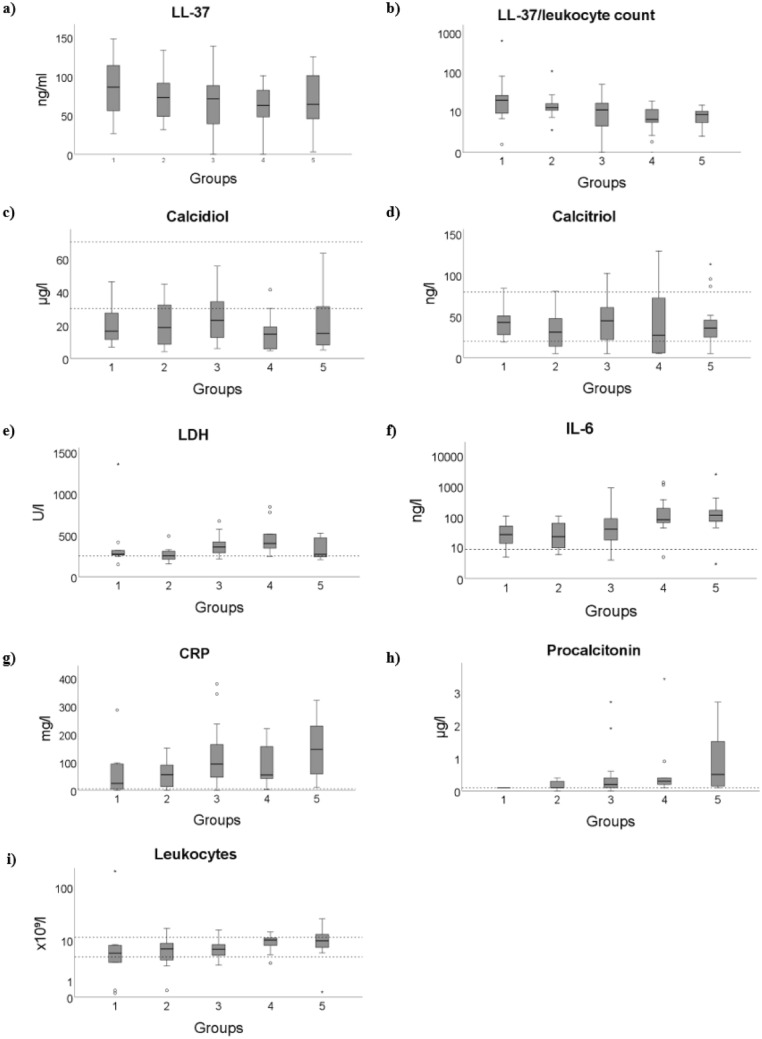
Boxplot of parameters significantly correlated with COVID-19 severity. The median, upper quartile, lower quartile, minimum and maximum are shown. The dashed lines show the reference values. The correlation coefficients and significances are shown in Table 2. Calidiol and calcitriol are not significantly correlated with COVID-19 severity.

LL-37 binds to the spike protein of SARS-CoV-2 and may prevent the virus from entering the host cell^9^. We assumed a protective effect of LL-37 in COVID-19 and a relationship between the LL-37 serum levels and COVID-19 severity. Therefore, we examined LL-37 serum levels in our patients. To date, no defined LL-37 reference values exist. In our study, serum LL-37 levels ranged from 1 to 148 ng/ml, with an average serum level of 68 ng/ml. We found a decrease in mean LL-37 serum levels from 87 ng/l in group 1 to 57 ng/l in group 4. However, there was no statistically significant correlation between LL-37 serum levels and the severity of COVID-19, age, or sex of patients (Fig. 2a).

### LL-37 and leukocytes

LL-37 is produced in epithelial cells and neutrophil granulocytes. We therefore examined the ratio of serum LL-37 levels as a function of leukocyte count. The ratio indicates how much LL-37 is produced per leukocyte. We found a highly significant inversely proportional correlation between the LL-37/leukocyte count ratio and the severity of COVID-19 (*p* < 0.001) (Fig. 2b). The asymptomatic patients had the highest ratio with a mean of 81, whereas the deceased group (group 5) and the mechanically ventilated patients (group 4) had the lowest ratios with a mean of 8 (Table 1). In contrast, LL-37 serum levels as function of lymphocyte count did not correlate with COVID-19 severity (Supplementary Fig. 1b).

### Vitamin D

A recent study showed an association between vitamin D and COVID-19 severity^13^. Vitamin D regulates calcium balance and modulates immune system activity. A distinction is made between the precursor of vitamin D, calcidiol, and the active vitamin D hormone, calcitriol. In our study, mean serum calcidiol levels were below the reference range of 30 – 70 µg/l in all groups, whereas mean serum calcitriol levels were within the reference range of 20–79 ng/l (Table 1). Serum calcidiol and calcitriol levels showed no correlation with COVID-19 severity (Fig. 2c,d). Vitamin D has a stimulatory effect on LL-37^3^, so we also examined the relationship between these concentrations. We found no significant association between serum vitamin D and LL-37 levels (Supplementary Fig. 2a,b).

### Inflammatory markers CRP, IL-6, and PCT

The inflammatory response plays a crucial role in COVID-19. We therefore determined serum levels of the inflammatory markers IL-6, CRP, and procalcitonin. CRP is an acute-phase plasma protein produced in the liver. Serum CRP levels above 100 mg/l are indicative of systemic high-grade inflammation. In all groups, the mean serum CRP level was above the reference value of 5.0 mg/l. While groups 1 and 2 had the lowest increase in serum CRP level, the value was highest in group 5 (Table 1). We found a positive correlation between serum CRP level and the severity of COVID-19 (*p* = 0.03) (Fig. 2g).

IL-6 is a proinflammatory cytokine and a marker of inflammatory processes. Serum IL-6 levels above 150 ng/l are indicative of systemic high-grade inflammation. The mean serum IL-6 level was above the reference value of 8 ng/l in all five groups (Table 1). We found a positive correlation between the serum concentration of IL-6 and the severity of disease progression (*p* < 0.001) (Fig. 2f).

The detection of sepsis associated with COVID-19 is of great prognostic and therapeutic importance. Therefore, we determined serum PCT levels. Serum PCT levels above 2 µg/ml indicate systemic bacterial infection and are considered a sign of sepsis. The serum PCT value in group 5 showed the highest increase with a mean value of 0.95 µg/l (Table 1). COVID-19 severity correlated positively with serum PCT levels (*p* = 0.002) (Fig. 2h).

### Lactate dehydrogenase (LDH)

LDH is an enzyme found in all human cells. The serum LDH level correlates with COVID-19 mortality^14^. An increase in serum LDH level above 250 U/l indicates cell death and tissue destruction. Mean serum LDH levels were higher than the reference value in all groups, with the highest mean level in group 4 (Table 1). We found a positive correlation between serum LDH levels and COVID-19 severity (*p* = 0.032) (Fig. 2e).

### Leukocyte and lymphocyte count

A correlation between white blood cell count and the severity of COVID-19 has been noted in the literature^11^. A change in white blood cell count can have several causes. Bacterial and viral infectious diseases such as COVID-19 can cause both an increase (leukocytosis) and a decrease (leukopenia) in white blood cells. In our study, 18 patients had leukocytopenia, in which the white blood cell count was below the reference value of 4.4 × 10^3^ cells/µl. 15 patients had leukocytosis with a white blood cell count above the upper reference value of 11.3 × 10^3^ cells/µl. The white blood cell count correlated positively with COVID-19 severity (*p* = 0.02) (Fig. 2i).

A change in lymphocyte count may have multiple causes, including viral infectious diseases such as COVID-19, so we determined absolute lymphocyte counts. Overall, 53% of patients had absolute lymphocytopenia (< 1.2 × 10^9^ cells/l). There was no significant difference between the groups (Supplementary Fig. 1a).

### Immunoglobulins (Ig)

A COVID-19 infection causes the synthesis of specific immunoglobulins against SARS-CoV-2^15^. In this study, we investigated a correlation between total IgM, IgA, and IgG serum levels and the severity of COVID-19. Mean serum IgM, IgA, and IgG levels were within the reference range in all 5 groups. Serum IgM, IgA, and IgG levels showed no correlation with COVID-19 severity (Supplementary Fig. 1c–e).

### Liver function

Liver disease may also occur with COVID-19^16^. Therefore, we examined ASAT, ALAT, and gamma-GT, the standard biomarkers of liver injury. We found an increase in mean serum ASAT, ALAT, and gamma-GT levels above the reference range (Table 1). There was no significant difference between the groups (Supplementary Fig. 1f–k).

### Renal function

There is evidence of an association between COVID-19 mortality and impaired renal function^17^. Creatinine is a reliable marker for renal function. The mean serum creatinine level was above the reference ranges in the groups, except for groups 1 and 2 (female) and group 3 (male) (Table 1). There was no correlation between creatinine concentration and COVID-19 severity (Supplementary Fig. 1l–m).

### Lipoproteins

LL-37 has been shown to bind to various lipids^18^. The binding of LL-37 to lipoproteins represents an important reservoir for LL-37 and can influence serum LL-37 levels. Therefore, we examined the relationship between serum LL-37 and serum lipids. We did not find any correlation between serum LL-37 levels and serum lipid levels (Supplementary Fig. 1r–v).

Elevated total cholesterol, triglyceride, and LDL levels are important risk factors in the development of cardiovascular disease. In contrast, high HDL levels have a beneficial effect. We also examined the association between serum lipids and COVID-19 severity. Mean serum triglyceride, cholesterol, and LDL levels were within the reference range in all groups, whereas mean serum HDL levels were below the reference value in each group. We did not detect any correlation between serum lipid levels and COVID-19 severity (Supplementary Fig. 1r–v).

### Apolipoproteins

Apolipoproteins are the protein component of lipoproteins. Moreover, apolipoprotein A-I (apoA-I) causes inhibition of LL-37 in plasma and attenuates its cytotoxic effect, thereby protecting host cells^19,20^. Therefore, we examined apoA-I and apolipoprotein B (apoB) levels in serum. The mean serum apoA-I levels were below the reference value in groups 3, 4, and 5. Mean serum apoB levels were within the reference range in all groups (Table 1). Serum levels of apoA-1 and apoB did not correlate with COVID-19 severity (Supplementary Fig. 1n–q). There was no correlation between LL-37 serum levels and apoA-I and apoB serum levels.

## Discussion

Studies have described the beneficial effect of vitamin D on the disease course of COVID-19, although randomized controlled trials are still pending^21–23^. Cathelicidin, the precursor of LL-37, is strongly upregulated by vitamin D^24,25^. In addition, LL-37 binds with high affinity to the SARS-CoV-2 spike protein, preventing the spike protein from binding to the host cell^9^. LL-37 also binds to host cell ACE2 and prevents the spike protein from binding to the host cell, thereby preventing virus entry^9^. Those studies led us to believe that serum LL-37 levels and COVID-19 severity might be related. Therefore, we examined the serum LL-37 levels of COVID-19 patients. We found a decrease in mean LL-37 serum levels from 87 ng/l in group 1 to 57 ng/l in group 4 (Table1). Surprisingly, this difference was not statistically significant and there was no statistically significant correlation between serum LL-37 levels and COVID-19 severity.

A recent study compared serum cathelicidin levels of healthy persons and COVID-19 patients^26^. It found a significant difference between healthy and SARS-CoV-2 infected persons but no correlation between serum cathelicidin levels and COVID-19 severity. In our study, we examined serum levels of free (unbound) LL-37. LL-37 is known to bind to lipids and apolipoproteins^18,19^. Because LL-37 is cytotoxic to human cells at higher concentrations, binding of LL-37 to lipids and apolipoproteins may regulate serum levels of LL-37 and help avoid cytotoxicity^19,27^. In addition, the binding of LL-37 may serve as an LL-37 reservoir for rapid LL-37 accumulation in the event of infection. LL-37 is produced by various cells, such as epithelial cells, and by white blood cells in response to infection and after stimulation with vitamin D^28,29^. Hence, serum LL-37 levels are also dependent on leukocyte count, and leukocyte count varies during a COVID-19 infection. Therefore, we calculated serum LL-37 levels as a function of leukocyte count. The ratio indicates how much LL-37 is produced per leukocyte. LL-37 is part of the innate immune system, and the release of LL-37 and the LL-37/leukocyte count ratio are indicators of the activity of the innate immune system. We found a highly significant inversely proportional correlation between the LL-37/leukocyte count ratio and the severity of COVID-19. Since blood samples had been collected at the beginning of the inpatient stay, the LL-37/leukocyte count ratio could indicate a greater likelihood of a severe course of COVID-19 early. In our study population, the mean LL-37/leukocyte count ratio was less than 10 in the mechanical ventilated and deceased patients (groups 4 and 5, Table 1). Our results provide evidence that the LL-37/leukocyte count ratio could be used as a marker for early prognosis of the COVID-19 severity.

Because white blood cells are recruited from vessels to extravascular inflamed tissues, local extravascular concentrations of LL-37 may differ from serum LL-37 levels. In addition, LL-37 is released by neutrophil degranulation, and the local LL-37 concentration may be much higher^30^. During the course of COVID-19, some patients develop severe pneumonia, and leukocytes are recruited to the airways and lungs^31^. Granulocytes and macrophages locally secrete large amounts of LL-37, and local LL-37 levels in the airways and lungs may differ from serum LL-37 levels. Examination of LL-37 concentrations in the broncho-alveolar lavage fluid (BALF) of COVID-19 patients may reveal a correlation between LL-37 levels and COVID-19 severity. The presence of LL-37 in BALF is mainly due to the degranulation of neutrophil granulocytes. This process means that activated leukocytes are surrounded by a gradient of cathelicidins at the site of infection. Because pneumonia in COVID-19 patients is associated with massive mucus production with leukocytes^32,33^, it is likely that high concentrations of LL-37 are present in the BALF of COVID-19 patients. As a comparison, the LL-37 concentration of 30 µg/ml in BALF of cystic fibrosis patients is sixfold higher than an LL-37 concentration of 5 µg/ml in BALF of healthy individuals^34^. In addition, a significant correlation has been found between the concentration of LL-37 in nasal secretions and the severity of acute bronchiolitis in hospitalized infants and young children^35^.

COVID-19 has a mortality rate that is currently higher in northern latitudes above 35°^36^. Latitude 35° north is the latitude above which most people do not receive sufficient sunlight in winter to maintain adequate vitamin D levels^36^. Vitamin D could thus have a beneficial effect on COVID-19 severity. Therefore, we examined the serum vitamin D levels of COVID-19 patients in relation to the severity of COVID-19. We found no correlation between serum vitamin D levels and COVID-19 severity. Some studies support our findings but others do not. A recent study showed that low calcidiol levels in hospitalized patients with COVID-19 were associated with severe disease and increased ICU admission and mortality^13^. The results of a pilot study in Spain suggest that vitamin D supplementation may reduce disease severity^52^. In contrast, a retrospective cohort study of 231 patients found that serum calcidiol levels were not significant in predicting mortality in patients with SARS-CoV-2^37^. Another cohort study suggested a similar risk of mortality in COVID-19, independent of vitamin D concentration^38^. Further interventional studies are needed on the potential beneficial effect of vitamin D on COVID-19 severity.

Vitamin D has been shown to upregulate the CAMP gene encoding LL-37 and stimulate LL-37 production^39^. This upregulation suggests a positive relationship between serum vitamin D and serum LL-37 levels. Nevertheless, we found no correlation between serum vitamin D and serum LL-37 levels. Again, this finding may be explained by the binding of LL-37 to serum lipids and apolipoproteins, or because we measured free (unbound) serum LL-37 levels.

Measurement of calcidiol, the precursor of the vitamin D hormone (calcitriol), is recommended for assessing vitamin D deficiency. In our study, all 5 patient groups showed a deficiency of calcidiol. In contrast, mean calcitriol concentrations were within the normal range (Table 1). In recent years, vitamin D deficiency has been very present in the media in Germany, and calcitriol intake is common. Because we did not ascertain whether our patients had taken calcitriol supplements before their hospitalization, we cannot exclude a supplemental calcitriol intake as a reason for the calcidiol deficiency and the normal serum calcitriol levels of our study population. However, the discrepancy could arise from inaccuracy in the current reference values for calcidiol and calcitriol. New studies may be needed to determine the validity of the stated reference values.

Studies have already shown an association between age and COVID-19 severity. The older the patient, the higher the risk of a more severe course of COVID-19 and mortality^40,41^. In general, older patients have a weaker immune system and are more likely to have preexisting conditions than younger patients. Surprisingly, we found no correlation between age and COVID-19 severity (Table 2). However, the mean age of all our groups was comparatively high, ranging between 52 and 68 (Table 1). Potentially, older asymptomatic COVID-19 patients or older COVID-19 patients with mild symptoms were hospitalized out of caution, in contrast to younger COVID-19 patients, who were not concerned about a severe course of COVID-19 and did not go to the hospital.

Determination of inflammatory markers may be helpful in assessing the severity and prognosis of COVID-19. CRP is elevated in acute inflammatory processes, which are common in SARS-CoV-2 infections. We found a positive correlation between serum CRP levels and COVID-19 severity (Table 2). We found the highest average serum CRP levels in patient groups 3, 4, and 5 (Table 1). All patients in those groups required ventilation and some died from COVID-19. In particular, the patients who died, group 5, had the highest serum CRP levels, with a mean serum CRP value of more than 156 mg/l, indicating high-grade systemic inflammation. A recent study showed similar results and that high serum CRP levels were associated with a more severe disease course in COVID-19 patients^42^. Our results confirm that serum CRP level is an important marker for early detection of severe COVID-19 disease and timely intensive medical intervention.

In our study, COVID-19 severity correlated positively with serum levels of IL-6 (Table 2), and groups 4 and 5 had mean serum IL-6 levels above 150 ng/l (Table 1). Serum IL-6 levels above 150 ng/l are indicative of severe inflammation. A recent study shows that serum IL-6 levels can effectively assess the severity of COVID-19 and predict disease progression in patients^43^. That finding confirms our findings and highlights the importance of serum IL-6 as an indicator of disease severity. Regular monitoring of inflammatory parameters IL-6 and CRP is recommended^44^ and clinical studies assessed a possible therapeutic benefit of an IL-6 receptor blockade in COVID-19^45,46^.

We demonstrated a positive correlation between serum PCT levels and COVID-19 severity (Table 2). A recent study demonstrated higher serum PCT levels in patients who required intensive care^47^. In our study, the mean serum PCT concentration was less than 2.0 µg/l in all groups (Table 1). Values above 2.0 µg/l indicate systemic bacterial infection. Values between 0.5 and 2.0 µg/l are considered pathological but are in the borderline range where systemic, bacterial infection can neither be reliably diagnosed nor excluded. In another study, PCT concentrations between 2 µg/l and 0.5 µg/l were detected in COVID-19 patients^43^. Thus, concurrent bacterial involvement in addition to infection with SARS-CoV-2 may occur but does not seem to be the rule.

Pneumonia and pulmonary fibrosis are common findings in severe COVID-19 cases^14^ and are associated with tissue destruction and cell death. Studies have shown that an increase in serum LDH level is associated with severe COVID-19 and increased mortality^14,48^. In our study, the mean serum LDH level was above the reference range of 250 U/l in all 5 groups. We found a significant correlation between serum LDH levels and COVID-19 severity. In a previous study, we demonstrated an increase in serum LDH levels in moderate and severe courses of COVID-19 above 400 U/l^48^. In the current study, only group 4 achieved a comparably high mean serum LDH level. The mean serum LDH levels of the other groups were below 400 U/l. The difference in serum LDH levels between the two studies may be explained by improved treatment options. Treatment with dexamethasone, which has an anti-inflammatory effect, has been shown to reduce the extent of ensued airway and lung inflammation and mortality in COVID-19 patients requiring oxygen supplementation^49^.

In addition to markers of inflammation, the white blood cell count can also be an important indicator of infections. Leukocytopenia can occur when the consumption of leukocytes to ward off the infection outweighs the new synthesis of leukocytes in the bone marrow. Leukocytosis can be caused by lymphocytosis or granulocytosis as a cytokine-induced immune response to viral or bacterial infection. The COVID-19 patients of our study had both leukocytopenia and leukocytosis. The white blood cell count correlated positively with COVID-19 severity. Our findings are in line with a recent study that found a positive correlation between white blood cell count and disease severity^11^: Patients with a severe course of COVID-19 had leukocytosis more frequently than patients with a mild course^11^. Recently, the white blood cell count of 1099 Chinese patients with COVID-19 was investigated and leukopenia was found in 34% of the cases^50^. These results support our findings that both leukocytopenia and leukocytosis can occur in patients with COVID-19.

In our study, in addition to CRP, IL-6, LDH, and leukocyte count, we identified the ratio of LL-37 to leukocyte count as another prognostic laboratory parameter that correlates with COVID-19 severity. All parameters were determined on days 1–3 after hospitalization. Severity grading was performed after recovery from COVID-19 or patient death. All parameters, CRP, IL-6, LDH, leukocyte count, and LL-37/leukocyte count, can be considered early prognostic parameters. Currently, no decision limits have been defined for the parameters, e.g., limits above which CRP concentration or below which LL-37/leukocyte ratio patients will die. Without decision limits, it is not possible to assess which parameter correctly predicts the course of COVID-19 more often. Therefore, their prognostic significance cannot be compared. Further, each parameter has a different meaning. CRP and IL-6 are inflammatory markers reflecting the extent of the systemic proinflammatory response. Leukocytes play a central role in defense against infection and leukocyte production is generally stimulated during infection. However, it can also decrease due to consumption during the course of infection. LDH, in turn, reflects the extent of cell and tissue damage. LL-37 is part of the innate immune system, and the release of LL-37 and the LL-37/leukocyte count ratio indicate the activity of the innate immune system. Because the parameters indicate different things, we advise considering all the parameters.

## Limitations

In our study, serum LL-37 levels of COVID-19 patients, but not of healthy subjects were compared. Therefore, the assessment of serum LL-37 levels is limited since no reference values for serum LL-37 exist. In particular, the comparison of serum LL-37 levels of healthy subjects with severely ill COVID-19 patients would have been interesting. Furthermore, the LL-37 ELISA detects LL-37 but also the precursor molecule human cathelicidin. Therefore, it is not possible to distinguish between active LL-37 and inactive human cathelicidin. However, human cathelicidin should be converted to LL-37 upon release^5^. Another limitation of our study is that the severity of COVID-19 disease was assessed without considering patients' preexisting conditions. A study suggest that preexisting conditions may have an impact on mortality^51^. In our study, blood samples were collected at the beginning of the inpatient stay. A longitudinal study with consecutive blood samples would provide further important insights. Furthermore, we did not differentiate between virus variants and subvariants. Our study was conducted before the delta-variant of SARS-CoV-2 became widespread.

## Conclusions

In our study, we found neither a significant correlation of LL-37 or vitamin D serum levels and severity of COVID-19 nor a significant correlation of vitamin D with LL-37 serum levels. However, we uncovered important aspects for investigating the relevance of LL-37 in the context of COVID-19 disease. LL-37 is produced by granulocytes and released at the site of inflammation. Therefore, the analysis of LL-37 in BALF rather than in serum seems critical. Since LL-37 is produced by granulocytes, we determined serum LL-37 levels as function of leukocyte count. The LL-37/leukocyte count ratio correlates highly significantly inversely proportional with the severity of COVID-19. Our results indicate that the progression of COVID-19 could be assessed using the LL-37/leukocyte count ratio as an early marker at the beginning of an inpatient stay.

## Supplementary Information

Supplementary Information.

## Data Availability

The datasets analysed during the current study are available from the corresponding author on reasonable request.
