# Supplementary material for: The ratio of serum LL-37 levels to blood leucocyte count correlates with COVID-19 severity
Source: Sci Rep. 2022 Jun 8;12:9447. doi: 10.1038/s41598-022-13260-8 (PMC9175165; doi:10.1038/s41598-022-13260-8)
Supplement: Supplementary file 1 — Supplementary Information. [file 41598_2022_13260_MOESM1_ESM.docx]

**The ratio of serum LL-37 levels to blood leucocyte count correlates with COVID-19 severity**

Matthias Keutmann^1^, Gabriele Hermes^1^, Denise Meinberger^1^, Annika Roth^1^, Jannik Stemler^2,3,4^, Oliver A. Cornely^2,3,4^, Andreas R. Klatt^1*^, and Thomas Streichert^1^

Supplementary Information

**Table S1: Sex and severity of COVID-19.** The table shows the sex distribution of the groups according to the severity of COVID-19.

|  | | **Groups** | | | | | **Total** |  |
| --- | --- | --- | --- | --- | --- | --- | --- | --- |
|  |  | **1** | **2** | **3** | **4** | **5** |  |  |
|  | **male** | 7 (70 %) | 6 (46 %) | 13 (50 %) | 8 (62 %) | 8 (50 %) | 42 (54 %) | |
|  | **female** | 3 (30 %) | 7 (54 %) | 13 (50 %) | 5 (39 %) | 8 (50 %) | 36 (46 %) | |


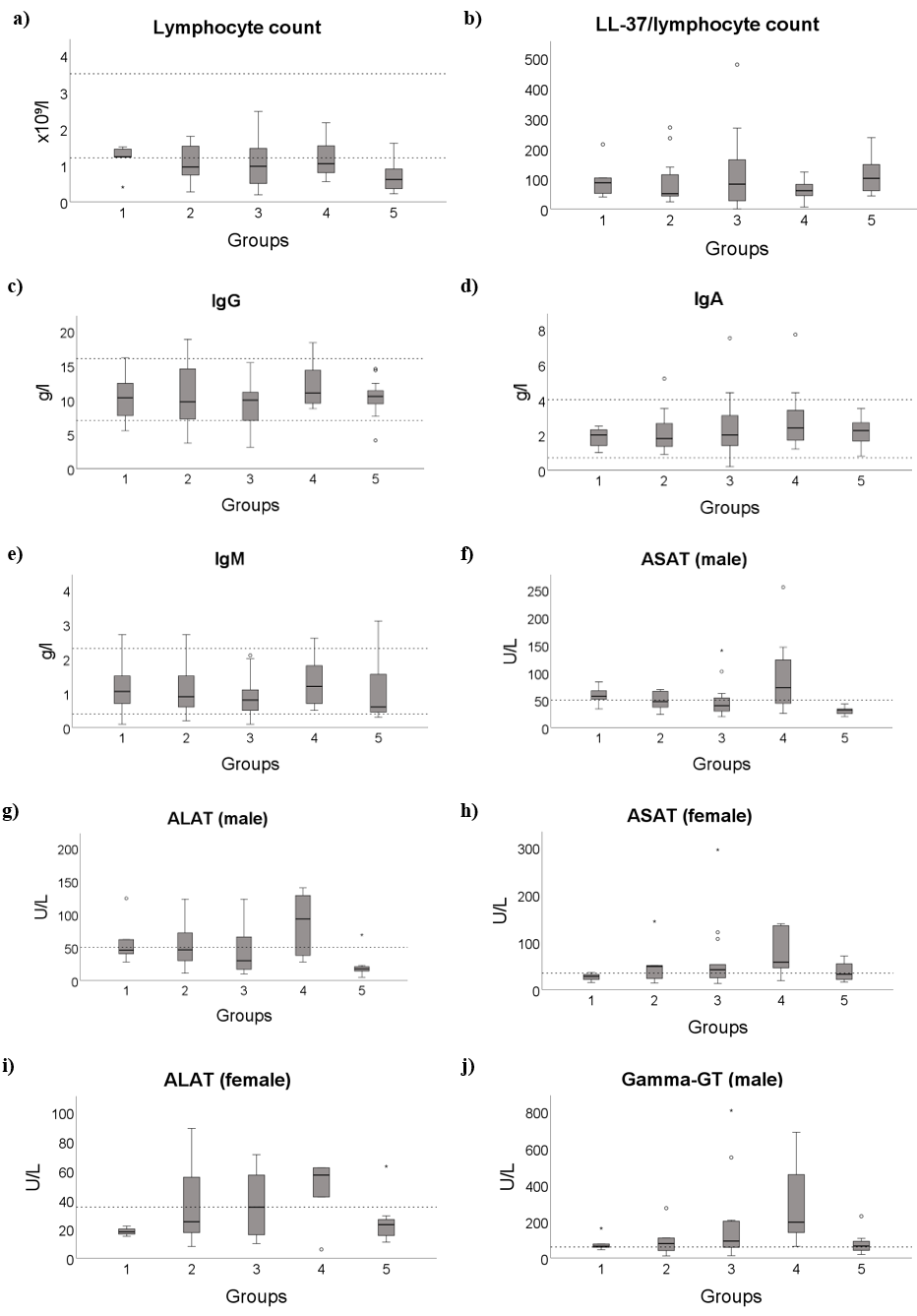


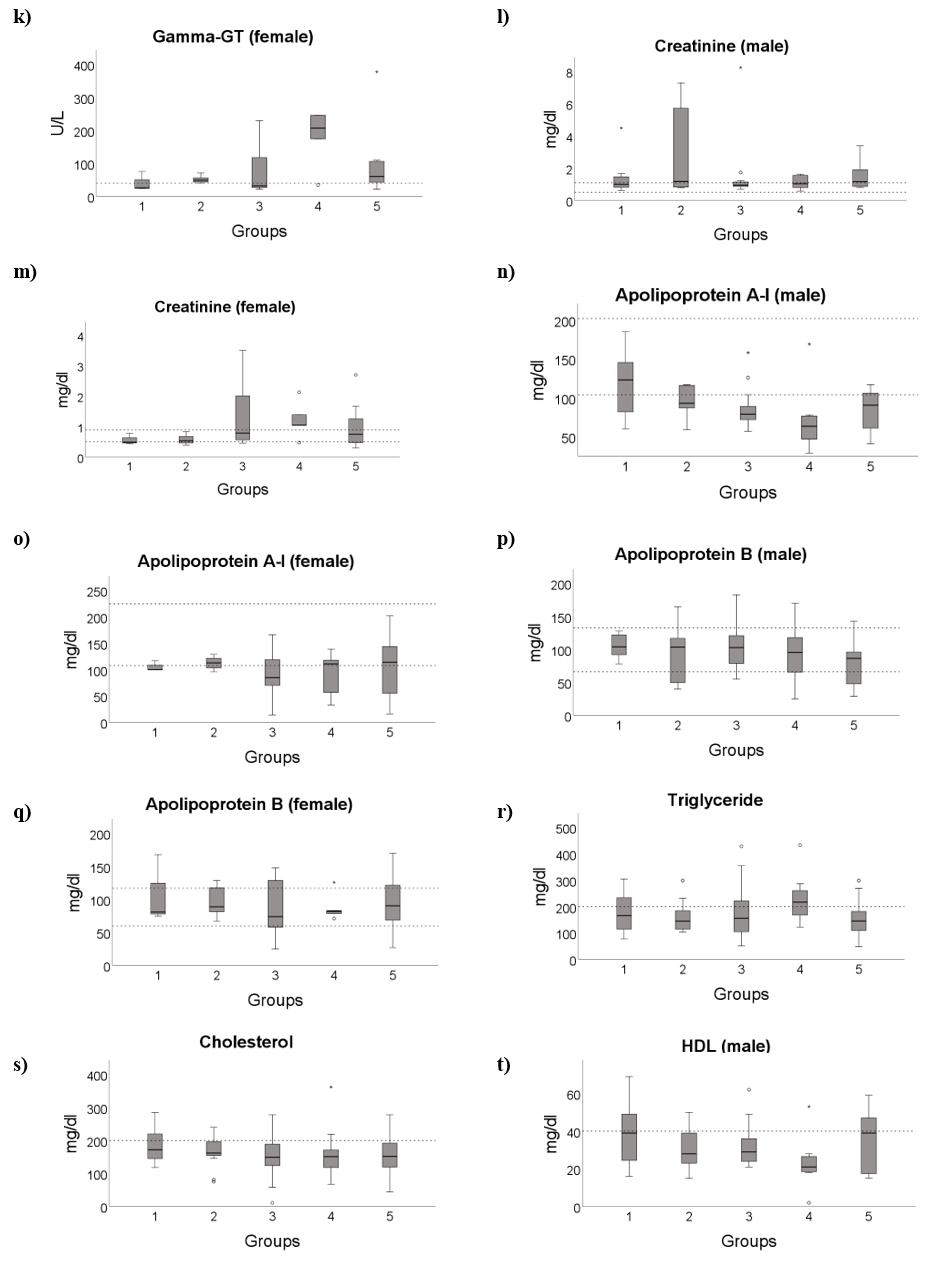


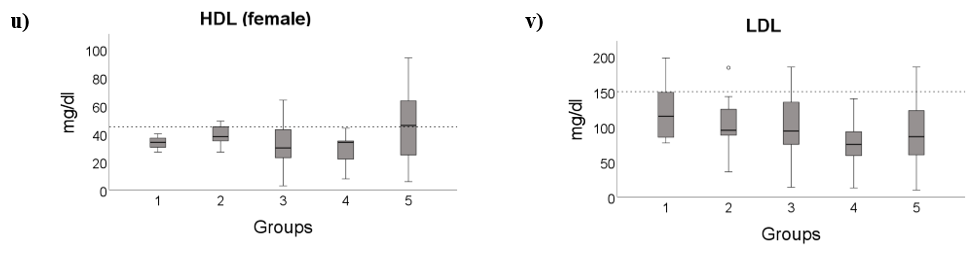


**Supplementary Figure S1: Correlations between laboratory parameters and COVID‑19 severity** The black lines are the regression grades; the dashed lines show the reference values. The correlation coefficients and significances are shown in Table 2.


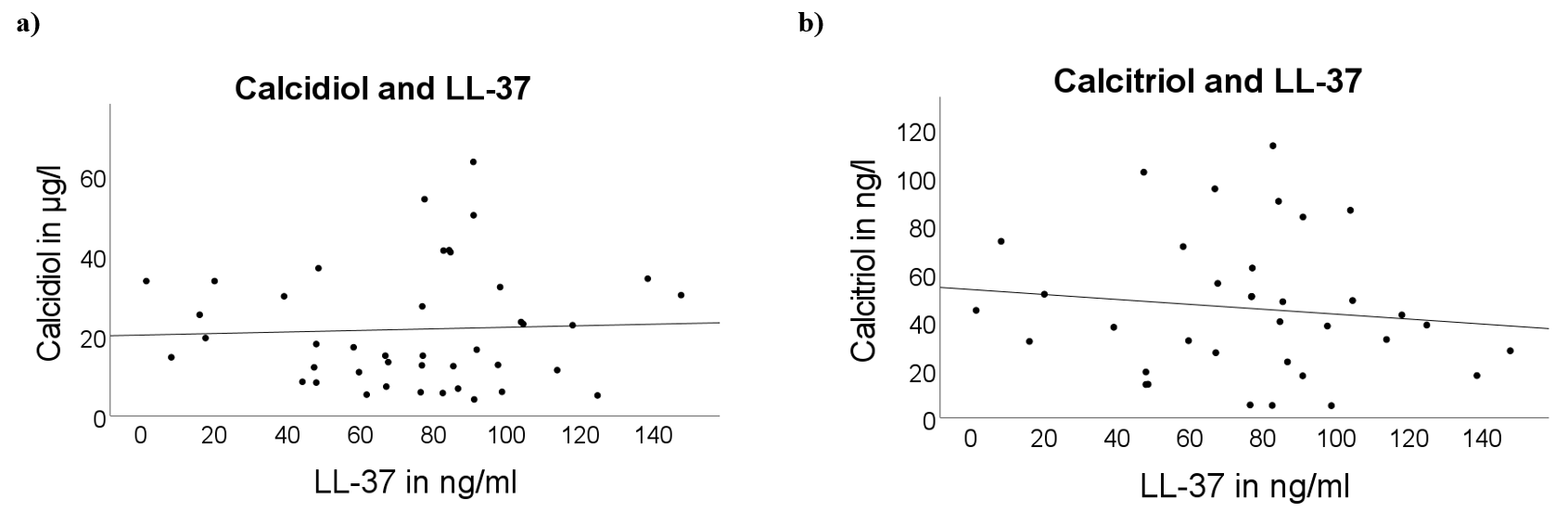


**Supplementary Figure S2: Correlations between vitamin D and LL-37.** The black lines are the regression grades; the dashed lines show the reference values. The correlation coefficients and significances are shown in Table 2.
